# Risk of Stroke and Post-Stroke Adverse Events in Patients with Exacerbations of Chronic Obstructive Pulmonary Disease

**DOI:** 10.1371/journal.pone.0169429

**Published:** 2017-01-06

**Authors:** Chao-Shun Lin, Chun-Chuan Shih, Chun-Chieh Yeh, Chaur-Jong Hu, Chi-Li Chung, Ta-Liang Chen, Chien-Chang Liao

**Affiliations:** 1 Department of Anesthesiology, Taipei Medical University Hospital, Taipei, Taiwan; 2 Anesthesiology and Health Policy Research Center, Taipei Medical University Hospital, Taipei, Taiwan; 3 Department of Anesthesiology, School of Medicine, College of Medicine, Taipei Medical University, Taipei, Taiwan; 4 School of Chinese Medicine for Post-Baccalaureate, College of Medicine, I-Shou University, Kaohsiung, Taiwan; 5 Department of Surgery, China Medical University Hospital, Taichung, Taiwan; 6 Department of Surgery, University of Illinois, Chicago, Illinois, United States of America; 7 Department of Neurology, Shuang Ho Hospital, Taipei Medical University, New Taipei City, Taiwan; 8 Division of Pulmonary Medicine, Department of Internal Medicine, Taipei Medical University Hospital, Taipei, Taiwan; 9 School of Chinese Medicine, College of Chinese Medicine, China Medical University, Taichung, Taiwan; 10 Department of Anesthesiology, Shuan Ho Hospital, Taipei Medical University, New Taipei City, Taiwan; University of Palermo, ITALY

## Abstract

**Background:**

The risk and outcomes of stroke in patients with chronic obstructive pulmonary disease exacerbations (COPDe) remain unclear. We examined whether patients with COPDe faced increased risk of stroke or post-stroke outcomes.

**Methods:**

Using Taiwan’s National Health Insurance Research Database, we identified 1918 adults with COPDe and selected comparison cohorts of 3836 adults with COPD no exacerbations and 7672 adults without COPD who were frequency matched by age and sex in 2000–2008 (Study 1). Stroke event was identified during 2000–2013 follow-up period. Adjusted hazard ratios (HRs) and 95% confidence intervals (CIs) of stroke associated with COPDe were calculated. In a nested cohort study (Study 2) of 261686 new-diagnosed stroke patients in 2000–2009, we calculated adjusted odds ratios (ORs) and 95% CIs of adverse events after stroke in patients with COPDe.

**Results:**

Patients with COPDe had increased stroke incidence, with an adjusted HR of 1.28 (95% CI, 1.03–1.59). In the Study 2, COPDe were associated with post-stroke mortality (OR, 1.34, 95% CI 1.20–1.52), epilepsy (OR, 1.43; 95% CI, (1.22–1.67), and pneumonia (OR, 1.50; 95% CI, 1.39–1.62). Previous intubation for COPD and inpatient admissions due to COPD were factors associated with post-stroke adverse events.

**Conclusion:**

Patients who have had COPDe face increased risks of stroke and post-stroke adverse events.

## Introduction

Chronic obstructive pulmonary disease (COPD) was the fourth leading cause of death worldwide in 2008, and it is expected to move to third place by 2020 [1–3]. The worldwide prevalence of COPD (physiologically defined) is 3–11%, and approximately 4267500 COPD-related deaths occur in 2013 among 188 countries [4,5]. In United States, the medical costs attributable to COPD and its sequelae were estimated at $32.1 billion in 2010 and projected to reach $49 billion in 2020 [6].

Stroke was the second leading cause of death worldwide in 2010. It causes serious long-term disability and associated costs; in 2010 U.S. economic losses from stroke cost an estimated $73.7 billion [7]. A multicenter study reported ten leading risk factors that accounted for more than 90% of global stroke risk: cardiac disease, diabetes, alcohol intake, hypertension, current smoking, abdominal obesity, unhealthy diet, lack of exercise, psychosocial stress and depression [8]. Other risk factors associated with stroke require further study.

Previous studies have reported that patients with COPD no exacerbations (COPDne) or COPDe are at increased risk of stroke [9,10]. However, the risk of stroke for patients with COPDe is not completely understood. Previous research was limited by short-term follow-up, lack of adequate control group, and poor adjustment for potential confounders [9,10]. In addition, the impact of previous COPDe on mortality and complications after stroke remain unknown. Based on claims data from Taiwan’s National Health Research Database, we conducted two nationwide cohort studies to assess the risk of stroke and post-stroke mortality and complications in patients with COPDe.

## Methods

### Ethical approval

Reimbursement claims used in this study were collected from Taiwan’s National Health Insurance Research Database, which is available for research access [11–13]. To protect personal privacy, the database was decoded and patient identifications were scrambled for further public access for research. This study was evaluated and approved by the Joint Institutional Review Board of Taipei Medical University (TMU-JIRB-201605049) and E-DA Hospital (EDA-JIRB-2014012).

### Study design and population

We used Taiwan’s National Health Insurance Research Database to perform the current two studies. Source of data and details of this national database was described in our previous reports [11–13]. With using the one million representative sample of Taiwan’s National Health Insurance Research Database, we conducted a retrospective cohort study (Study 1) of 3634 patients with newly diagnosed COPDe (defined as COPD patients receiving emergency care or inpatient care, treated with steroid and/or antibiotics). With frequency matching by age and sex (case-control ratio = 1:2), 7268 patients with COPD (defined as at least two visits for outpatient care with physician’s primary diagnosis without exacerbations) were also identified as exposure cohort. For comparison, 14,536 frequency-matched (case-control ratio = 1:4) persons without COPD (no physician’s diagnoses of COPD) were selected. These three cohorts aged ≥20 years were established between January 1, 2000, and December 31, 2008, and then followed up until December 31, 2013. The inclusion criteria of COPDe were based on previous studies [10,14]. We calculated person-years during the follow-up period for each participant until the diagnosis of stroke or until being censored because of death, withdrawal from the insurance system, or loss to follow-up. The non-COPD group included the remaining people who did not experience COPD throughout follow-up.

In the stroke cohort from the National Health Insurance Research Database, consisting of all prevalent and incident stroke patients in 2000–2009 from the total population of 23 million people in Taiwan, we identified 261686 new-onset stroke patients with hospitalization (Study 2). There were 60854 stroke patients had previous COPD and 11,828 stroke patients had previous COPDe. We compared post-stroke acute myocardial infarction, gastrointestinal bleeding, epilepsy, pneumonia, urinary tract infection, medical expenditure, length of hospital stay, intensive care unit stay, and mortality for a 30-day period after stroke for stroke patients with pre-stroke COPDne, COPDe, and no COPD.

### Measures and definition

We identified income status by defining low-income patients as those qualifying for waived medical copayment verified by Taiwan Bureau of National Health Insurance. Whether the surgery was performed in a teaching hospital and the types of surgery and anesthesia were also recorded. We used *the International Classification of Diseases*, *Ninth Revision*, *Clinical Modification* (ICD-9-CM) to define chronic obstructive pulmonary disease (ICD-9-CM 491, 492, 496), stroke (ICD-9-CM 430–438), preoperative medical diseases, and post-stroke complications. Coexisting medical conditions that were determined from medical claims for the 24-month pre-stroke period included hypertension (ICD-9-CM 401–405), mental disorders (ICD-9-CM 290–319), diabetes (ICD-9-CM 250), liver cirrhosis (ICD-9-CM 571), hyperlipidemia (ICD-9-CM 272.0, 272.1, and 272.2), congestive heart failure (ICD-9-CM 428), traumatic brain injury (ICD-9-CM 800–804, 850–854), anemia (ICD-9-CM 280–285), atrial fibrillation (ICD-9-CM 427.31), and peripheral vascular disease (ICD-9-CM 443). Renal dialysis was identified by administration code (D8, D9). People who visited outpatient care for obesity and smoking cessation were identified in this study. In-hospital 30-day mortality after the index surgery was considered the primary outcome, and post-stroke epilepsy (ICD-9-CM 345), pneumonia (ICD-9-CM 480–486), acute myocardial infarction (ICD-9-CM 410), gastrointestinal bleeding (ICD-9-CM 578), urinary tract infection (ICD-9-CM 599.0) were considered secondary outcomes in the nested cohort study. Length of hospital stay during stroke admission was also analyzed.

### Statistical analyses

We compared sociodemographic factors (such as age, sex, and low income), coexisting medical conditions (such as hypertension, mental disorders, diabetes, liver cirrhosis, hyperlipidemia, congestive heart failure, traumatic brain injury, anemia atrial fibrillation, peripheral vascular disease, renal dialysis, epilepsy, obesity, and smoking cessation), and medications (such as anticoagulant, anti-platelet agents, and lipid-lowering agents), for people with COPDne, with COPDe, or with no COPD using the chi-square tests. The adjusted hazard ratios (HRs) and 95% confidence intervals (CIs) of stroke associated with COPDe were calculated by using multivariate Cox proportional hazard models. In the further stratified analysis, the adjusted HRs and 95% CIs of stroke in patients with COPDne and COPDe were also calculated in both sexes and all age groups.

In the nested cohort study, the chi-square tests were used to compare differences relating to sociodemographics, coexisting medical conditions, and medications between stroke patients without COPD and with previous COPDe. By using multivariate logistic regressions, we calculated adjusted odds ratios (ORs) and 95% CIs of the risks of adverse events after stroke, including acute myocardial infarction, gastrointestinal bleeding, epilepsy, pneumonia, urinary tract infection, increased medical expenditure, prolonged length of stay in intensive care unit, and mortality for stroke patients with previous COPDne and COPDe.

## Results

After matching by age and sex among cohorts with COPDne, COPDe and without COPD (Table 1), patients with COPDe showed higher proportions of having low-income status, hypertension, mental disorders, diabetes, liver cirrhosis, hyperlipidemia, congestive heart failure, traumatic brain injury, anemia, atrial fibrillation, and peripheral vascular disease than people without COPD (all p<0.0001). Use of medications such as anticoagulant, anti-platelet agents, lipid-lowering agents, methylxanthines, beta2-adrenergic agonist, systemic corticosteroids, anticholinergics, and inhaled corticosteroids were also higher in patients with COPDe than in those without COPD (all p<0.0001).

**Table 1:** Sociodemographic factors and coexisting medical conditions in people with and without COPD.

	No COPD N = 7672		COPDne N = 3836		COPDe N = 1918		*P* value^a^	*P* value^b^
Sex	n	(%)	n	(%)	n	(%)	1.0000	1.0000
Female	2800	(36.5)	1400	(36.5)	700	(36.5)		
Male	4872	(63.5)	2436	(63.5)	1218	(63.5)		
Age, years							1.0000	1.0000
40–49	1712	(22.3)	856	(22.3)	428	(22.3)		
50–59	2664	(34.7)	1332	(34.7)	666	(34.7)		
60–69	2240	(29.2)	1120	(29.2)	560	(29.2)		
≥70	1056	(13.8)	528	(13.8)	264	(13.8)		
Low income	128	(1.7)	115	(3.0)	116	(6.1)	<0.0001	<0.0001
Medical conditions								
Hypertension	3133	(40.8)	1969	(51.3)	990	(51.6)	<0.0001	0.8374
Mental disorder	2120	(27.6)	1747	(45.5)	922	(48.1)	<0.0001	0.0698
Diabetes	1585	(20.7)	907	(23.6)	511	(26.6)	<0.0001	0.0129
Liver cirrhosis	921	(12.0)	801	(20.9)	346	(18.0)	<0.0001	0.0110
Hyperlipidemia	1061	(13.8)	746	(19.5)	274	(14.3)	<0.0001	<0.0001
Congestive heart failure	252	(3.3)	278	(7.3)	282	(14.7)	<0.0001	<0.0001
Traumatic brain injury	410	(5.3)	299	(7.8)	241	(12.6)	<0.0001	<0.0001
Anemia	417	(5.4)	322	(8.4)	145	(7.6)	<0.0001	0.2747
Atrial fibrillation	95	(1.2)	106	(2.8)	81	(4.2)	<0.0001	0.0032
PVD	166	(2.2)	161	(4.2)	58	(3.0)	<0.0001	0.0284
Renal dialysis	123	(1.6)	69	(1.8)	39	(2.0)	0.3920	0.5364
Epilepsy	59	(0.8)	50	(1.3)	33	(1.7)	0.0003	0.2110
Obesity	32	(0.4)	23	(0.6)	14	(0.7)	0.0003	0.5598
Smoking cessation	7	(0.1)	8	(0.2)	7	(0.4)	0.0215	0.2727
Number of medical conditions							<0.0001	0.0074
0	2310	(30.1)	618	(16.1)	287	(15.0)		
1	2326	(30.3)	994	(25.9)	494	(25.8)		
2	1637	(21.3)	931	(24.3)	425	(22.2)		
3	889	(11.6)	695	(18.1)	342	(17.8)		
≥4	510	(6.7)	598	(15.6)	370	(19.3)		
Anticoagulant	241	(3.1)	208	(5.4)	161	(8.4)	<0.0001	<0.0001
Anti-platelet agents	3340	(43.5)	2353	(61.3)	1234	(64.3)	<0.0001	0.0269
Lipid-lowering agents	2252	(29.4)	1464	(38.2)	712	(37.1)	<0.0001	0.4419
Methylxanthines	85	(1.1)	499	(13.0)	596	(31.1)	<0.0001	<0.0001
Beta2-adrenergic agonist	2849	(37.1)	2750	(71.7)	1686	(87.9)	<0.0001	<0.0001
Systemic corticosteroids	439	(5.7)	977	(25.5)	1200	(62.6)	<0.0001	<0.0001
Anticholinergics	3712	(48.4)	3250	(84.7)	1732	(90.3)	<0.0001	<0.0001
Inhaled corticosteroids	3812	(49.7)	2878	(75.0)	1623	(84.6)	<0.0001	<0.0001

COPD = chronic obstructive pulmonary disease; COPDe = chronic obstructive pulmonary disease exacerbations; COPDe = chronic obstructive pulmonary disease with no exacerbations. PVD = peripheral vascular disease.

^a^ Chi-square tests for people had no COPD, COPDne, and COPDe.

^b^ Chi-square tests between COPDne and COPDe.

**Fig 1:**
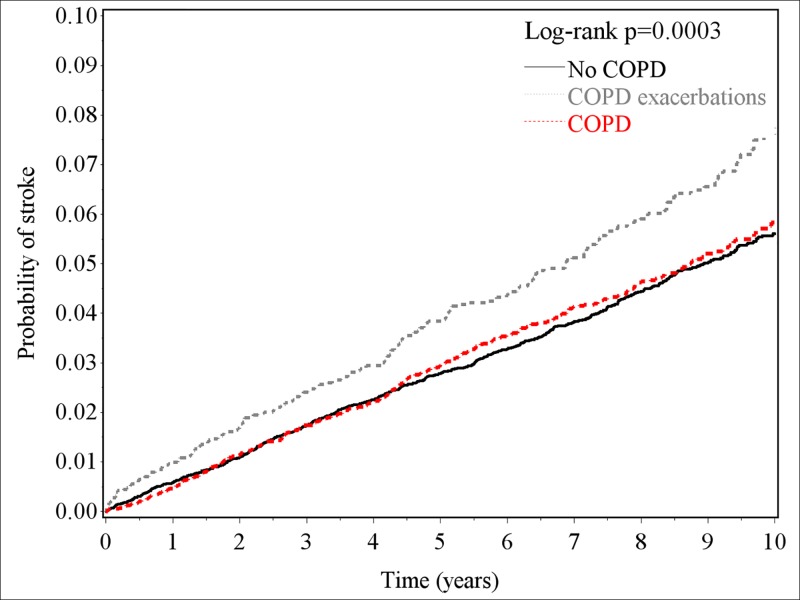
Kaplan-Meier curves for probabilities of having stroke in the study cohorts with COPDne, COPDe, and no COPD.

Table 2 shows that higher incidence of stroke was found in patients with previous COPDe than those without COPD (7.6 vs. 5.8 per 1000 person-years, p<0.0001) during the follow-up period. The corresponding HR of stroke associated with COPDe was 1.28 (95% CI, 1.03–1.59). The risk of stroke was not associated with COPDne during the follow-up period. The association between COPDe and stroke risk was significant in males (HR, 1.37; 95% CI, 1.06–1.77) and patients aged 50–69 years (HR, 1.44; 95% CI, 1.00–2.07). The probabilities of stroke at one, five and ten years after patients suffering COPDe were 1%, 4% and 8%, respectively (Fig 1). Compared with non-COPD cohort, patients with COPDe had increased probability of having stroke (log-rank test, p<0.0001), however, there was no significant difference between COPDne and non-COPD patients. The difference between non-COPD and COPDe increased with the follow-up time increased.

**Table 2:** Risk of stroke in association with previous COPD exacerbations by sex and age^b^.

	N	Events	PY	Incidence	HR (95% CI)^a^
No COPD	7672	385	65986	5.8	1.00 (reference)
COPDne	3836	194	34636	5.6	0.97 (0.88–1.06)
COPDe	1918	120	15812	7.6	1.28 (1.03–1.59)
Female					
No COPD	2800	131	25046	5.2	1.00 (reference)
COPDne	1400	58	12947	4.5	0.94 (0.80–1.10)
COPDe	700	34	6124	5.6	1.13 (0.76–1.68)
Male					
No COPD	4872	254	40939	6.2	1.00 (reference)
COPDne	2436	136	21688	6.3	0.98 (0.88–1.09)
COPDe	1218	86	9688	8.9	1.37 (1.06–1.77)
40–49 years					
No COPD	1712	39	16721	2.3	1.00 (reference)
COPDne	856	18	8434	2.1	0.85 (0.63–1.14)
COPDe	428	18	3932	4.6	1.29 (0.71–2.37)
50–59 years					
No COPD	2664	122	24162	5.0	1.00 (reference)
COPDne	1332	72	12362	5.8	1.04 (0.90–1.21)
COPDe	666	44	5858	7.5	1.44 (1.00–2.07)
60–69 years					
No COPD	2240	148	19046	7.8	1.00 (reference)
COPDne	1120	71	10153	7.0	0.96 (0.83–1.11)
COPDe	560	48	4597	10.4	1.37 (0.97–1.92)
≥70 years					
No COPD	1056	76	6057	12.5	1.00 (reference)
COPDne	528	33	3687	9.0	0.92 (0.75–1.13)
COPDe	264	10	1426	7.0	0.65 (0.33–1.30)

CI = confidence interval; COPD = chronic obstructive pulmonary disease; COPDe = chronic obstructive pulmonary disease exacerbations; COPDe = chronic obstructive pulmonary disease with no exacerbations; HR, hazard ratio; PY = person-years

^a^ Full model adjusted for all covariates listed in Table 1 except for respiratory medications.

^b^ Compared with non-COPD controls, the HR of hemorrhagic stroke, ischemic stroke, and other stroke for COPD patients without exacerbations were 0.87 (95% CI, 0.72–1.07), 0.97(95% CI, 0.87–1.09), 1.04(95% CI, 0.83–1.29), respectively; the corresponding HR for COPD patients with exacerbations were 1.42 (95% CI, 0.91–2.22), 1.27(95% CI, 0.97–1.66), and 1.09 (95% CI, 0.60–1.96), respectively.

Compared with patients without COPD (Table 3), stroke patients with previous COPDe had higher proportions of males, older people, low-income people, other stroke, stay in teaching hospital, hypertension, mental disorders, diabetes, congestive heart failure, traumatic brain injury, dementia, hyperlipidemia, anemia, atrial fibrillation, liver cirrhosis, peripheral vascular disease, renal dialysis, obesity, smoking cessation, anti-platelet agents, lipid-lowering agents, and anticoagulants, methylxanthines, beta2-adrenergic agonist, systemic corticosteroids, anticholinergics, and inhaled corticosteroids (all p<0.01).

**Table 3:** Characteristics of hospitalized stroke patients with and without COPD.

Number	No COPD		COPDne		COPDe		*P* value^a^	*P* value^b^
	230553		38997		7924			
Gender	n	(%)	N	(%)	n	(%)	<0.0001	<0.0001
Female	101287	(43.9)	13431	(34.4)	2071	(26.1)		
Male	129266	(56.1)	25566	(65.6)	5853	(73.9)		
Age, years							<0.0001	<0.0001
40–49	27378	(11.9)	1032	(2.7)	96	(1.2)		
50–59	50929	(22.1)	3186	(8.2)	362	(4.6)		
60–69	59344	(25.7)	7577	(19.4)	1074	(13.6)		
70–79	61397	(26.6)	16071	(41.2)	3314	(41.8)		
≥80	31505	(13.7)	11131	(28.5)	3078	(38.8)		
Low income	9701	(4.2)	2354	(6.0)	604	(7.6)	<0.0001	<0.0001
Types of stroke							<0.0001	<0.0001
Hemorrhage	48159	(20.9)	5603	(14.4)	1014	(12.8)		
Ischemia	142082	(61.6)	24313	(62.4)	4774	(60.3)		
Other	40312	(17.5)	9081	(23.3)	2136	(27.0)		
Stay in teaching hospital	210345	(91.2)	33339	(85.5)	6526	(82.4)	<0.0001	<0.0001
Medical conditions								
Hypertension	107302	(46.5)	22633	(58.0)	4430	(55.9)	<0.0001	0.0005
Mental disorder	34402	(14.9)	10402	(26.7)	2280	(28.8)	<0.0001	0.0001
Diabetes	55695	(24.2)	9661	(24.8)	1826	(23.0)	0.0015	0.0011
Congestive heart failure	5683	(2.5)	3385	(8.7)	1223	(15.4)	<0.0001	<0.0001
Traumatic brain injury	13100	(5.7)	3555	(9.1)	867	(10.9)	<0.0001	<0.0001
Dementia	6758	(2.9)	2623	(6.7)	687	(8.7)	<0.0001	<0.0001
Hyperlipidemia	15563	(6.8)	3340	(8.6)	454	(5.7)	<0.0001	<0.0001
Anemia	4996	(2.2)	1668	(4.3)	386	(4.9)	<0.0001	0.0185
Atrial fibrillation	2981	(1.3)	1241	(3.2)	330	(4.2)	<0.0001	<0.0001
Liver cirrhosis	2606	(1.1)	593	(1.5)	144	(1.8)	<0.0001	0.0529
PVD	2305	(1.0)	649	(1.7)	131	(1.7)	<0.0001	0.9442
Renal dialysis	4042	(1.8)	545	(1.4)	111	(1.4)	<0.0001	0.9820
Obesity	798	(0.4)	156	(0.4)	29	(0.4)	0.2494	0.6592
Smoking cessation	121	(0.1)	59	(0.2)	7	(0.1)	<0.0001	0.1728
Number of medical conditions							<0.0001	<0.0001
0	70929	(30.8)	7032	(18.0)	1422	(18.0)		
1	91344	(39.6)	13952	(35.8)	2612	(33.0)		
2	46512	(20.2)	10486	(26.9)	2142	(27.0)		
3	16322	(7.1)	5165	(13.2)	1162	(14.7)		
≥4	5446	(2.4)	2362	(6.1)	586	(7.4)	<0.0001	<0.0001
Lipid-lowering agents	40662	(17.6)	8084	(20.7)	1593	(20.1)	<0.0001	0.2090
Anti-platelet agents	110643	(48.0)	26503	(68.0)	5869	(74.1)	<0.0001	<0.0001
Anticoagulant	5008	(2.2)	1424	(3.7)	423	(5.3)	<0.0001	<0.0001
Methylxanthines	736	(0.3)	3122	(8.0)	1373	(17.3)	<0.0001	<0.0001
Beta2-adrenergic agonist	38200	(16.6)	23517	(60.3)	6932	(87.5)	<0.0001	<0.0001
Systemic corticosteroids	4121	(1.8)	9730	(25.0)	5219	(65.9)	<0.0001	<0.0001
Anticholinergics	63782	(27.7)	31115	(79.8)	7312	(92.3)	<0.0001	<0.0001
Inhaled corticosteroids	55318	(24.0)	20939	(53.7)	5954	(75.1)	<0.0001	<0.0001

COPD = chronic obstructive pulmonary disease; COPDe = chronic obstructive pulmonary disease exacerbations. PVD = peripheral vascular disease.

^a^ Chi-square tests for stroke patients had no COPD, COPDne, and COPDe.

^b^ Chi-square tests between patients with COPDne and patients with COPDe.

Table 4 shows that COPDne was associated with post-stroke epilepsy (OR, 1.27; 95% CI, 1.17–1.38), pneumonia (OR, 1.24; 95% CI, 1.18–1.29), and mortality (OR, 1.14; 95% CI, 1.07–1.22). Stroke patients with pre-stroke COPDe had higher risk of mortality (OR, 1.34; 95% CI, 1.20–1.52), epilepsy (OR, 1.43; 95% CI, 1.22–1.67), and pneumonia (OR, 1.50; 95% CI, 1.39–1.62) after stroke compared with those without COPD. Stroke patients with previous COPDne (p = 0.0048) or COPDe (p<0.0001) had longer hospital stays compared with stroke patients without COPD. Pre-stroke characteristics of COPDe (Table 5) such as experienced intubations (OR, 2.01; 95% CI, 1.89–2.15) and ≥3 visits for inpatient COPD care (OR, 1.66; 95% CI, 1.34–2.05) were significant factors associated with post-stroke adverse events. Similar situation was also found in patients with COPDne that experienced intubations (OR, 2.27; 95% CI, 2.19–2.36) and ≥3 visits for inpatient COPD care (OR, 1.66; 95% CI, 1.37–2.02) were risk factors for post-stroke adverse events.

**Table 4:** Risks of complications and mortality during stroke hospitalization associated with COPD and COPD exacerbations.

	No COPD		COPDne		COPDe		Risk of outcomes for COPDne	Risk of outcomes for COPDe
	n	(%)	n	(%)	n	(%)	OR (95% CI)^c^	OR (95% CI)^c^
Number	230553		38997		7924			
Mortality	7827	(3.4)	1373	(3.5)	344	(4.3)	1.14 (1.07–1.22)	1.34 (1.20–1.52)
Epilepsy	3786	(1.6)	798	(2.1)	192	(2.4)	1.27 (1.17–1.38)	1.43 (1.22–1.67)
Pneumonia	12877	(5.6)	3353	(8.6)	921	(11.6)	1.24 (1.18–1.29)	1.50 (1.39–1.62)
Acute myocardial infarction	1846	(0.8)	337	(0.9)	79	(1.0)	0.90 (0.80–1.02)	0.92 (0.72–1.16)
Gastrointestinal bleeding	5563	(2.4)	1087	(2.8)	245	(3.1)	0.98 (0.91–1.05)	0.97 (0.85–1.11)
Urinary tract infection	21071	(9.1)	4027	(10.3)	849	(10.7)	1.02 (0.98–1.06)	1.06 (0.98–1.14)
Adverse events^a^	22672	(9.8)	5051	(13.0)	1335	(16.9)	1.23 (1.19–1.28)	1.55 (1.42–1.63)
Length of stay, days^b^	8.6±7.0		8.5±6.8		9.3±7.1		0.0048	<0.0001

CI = confidence interval; COPD = chronic obstructive pulmonary disease; COPDe = chronic obstructive pulmonary disease exacerbations; OR = odds ratio.

^a^ Adverse events include pneumonia, epilepsy and mortality

^b^ Mean±SD

^c^ Adjusted for all covariates listed in Table 3 except for respiratory medications.

**Table 5:** Risk of post-stroke adverse events for patients with COPD exacerbations.

Pre-stroke characteristics of COPD	n	Adverse events after stroke^a^		
		Cases	(%)	OR (95% CI)^b^
No COPD	230553	22672	(9.8)	1.00 (Reference)
Stroke patients with COPDne (N = 38997)				
Had intubations	3126	1310	(41.9)	2.27 (2.19–2.36)
Had 1 visit for inpatient care	4030	650	(16.1)	1.33 (1.22–1.45)
Had 2 visits for inpatient care	885	162	(18.3)	1.53 (1.29–1.82)
Had ≥3 visits for inpatient care	668	130	(19.5)	1.66 (1.37–2.02)
Stroke patients with COPDe (N = 7924)				
Had intubations	1082	420	(38.8)	2.01 (1.89–2.15)
Had 1 visit for inpatient care	2606	392	(15.0)	1.20 (1.07–1.34)
Had 2 visits for inpatient care	692	134	(19.4)	1.61 (1.33–1.96)
Had ≥3 visits for inpatient care	563	110	(19.5)	1.66 (1.34–2.05)

CI = confidence interval; COPD = chronic obstructive pulmonary disease; COPDe = chronic obstructive pulmonary disease exacerbations; OR = odds ratio.

^a^ Adverse events include post-stroke pneumonia, epilepsy, and mortality

^b^ Adjusted for all covariates listed in Table 3 except for respiratory medications.

## Discussion

We conducted study 1 based on the claims data of Taiwan’s National Health Insurance system to analyze long-term risk of stroke in patients with COPDe during the follow-up period. Our Study 1 showed that COPDe is associated with significantly increased risk of stroke, while COPDne is not. The Study 2 showed that pre-stroke COPDe increased mortality and adverse events after stroke. Increased incidence of endotracheal intubation and hospitalization for COPD are significantly associated with post-stroke adverse events. This may be the first report verifying the impact of COPDe on risk of stroke and outcomes after stroke. The nationwide representative population, large sample size, cohort study design, and multivariate adjustment strengthen these two studies’ analytic power. In addition, our findings were similar with previous that COPD was more associated with hemorrhagic stroke than ischemic stroke [9,10].

Age, gender, and socioeconomic status were considered as potential confounding factors associated with COPD and stroke [3,15–18]. Considering the impact of COPDe on outcomes after stroke in the study 2, these characteristics need to be adjusted under the multivariate regression models. The present study showed approximately doubled stroke risk in patients with COPDe aged from 50 to 69, but not in patients aged 70 and higher. It is possible that older people have more medical illnesses, which may dilute the contribution of COPDe to risk of stroke. Another possible reason is that small size of stroke events occurred in patients with COPDe aged 40–49 years and ≥70 years lead to insignificances of statistical value.

Comorbidities including hypertension, mental disorder, diabetes, liver cirrhosis, hyperlipidemia, congestive heart failure, traumatic brain injury, anemia, atrial fibrillation, peripheral vascular disease, renal dialysis, epilepsy, and obesity were known factors associated with stroke [8,12,13,19–22]. These medical conditions also commonly coexisted in patients with COPD [1,23,24,25]. However, the previous study was limited by inadequate control of coexisting medical conditions, and this might cause confounding bias when estimating the risk of stroke in patients with COPDe [9,10]. To reduce these confounding effects, we used multivariate regression models to adjust coexisting medical conditions and calculate the risk and outcomes of stroke in patients with previous COPDe and patients with COPDne.

Stroke and COPDe have risk factors in common, such as smoking and low socioeconomic status. However, we suggest that increased inflammation is a plausible explanation for increased stroke risk in patients with COPDe. Most patients with COPDe were initially triggered by airway infection followed by systemic inflammation [26]. Previous studies have shown increased concentrations of fibrinogen and C-reactive protein during COPDe [27,28]. Elevated fibrinogen levels have been recognized as a predictor of stroke and marker of atherosclerosis [29]. C-reactive protein levels indicate underlying systemic inflammation and a novel plasma marker of atherothrombotic disease [30–32]. Elevated plasma C-reactive protein levels significantly predict the risk of ischemic stroke and transient ischemic attack [33]. Another possible explanation is that patients with COPDe are markedly inactive in daily life [34]. Physical activity lowers blood pressure and improves lipid profiles [35]. In addition, physical activity can also play an antithrombotic role by reducing blood viscosity [36], fibrinogen levels [37], and platelet aggregability [38], all of above might reduce stroke risk. Skeletal muscle dysfunction and wasting, which is commonly found in patients with COPDe, might play a role in reducing physical activity and therefore increase stroke risk [39,40]. An additional factor could be increased use of anticholinergics and β-agonists by the patient with COPDe. The former is believed to suppress parasympathetic control, whereas the latter is believed to stimulate sympathetic control, both causing an increased risk of tachyarrhythmias, stroke, and death [41,42]. Indeed, the effects of inflammation, inactivity, and tachyarrhythmias might be synergistic, while the underlined mechanisms need further investigation.

Medical complications are frequent after stroke. Pre-existing medical conditions, advanced age, and pre-stroke disability can affect an individual’s risk of developing post-stroke complications [43]. The current study showed that previous COPD exacerbations increased post-stroke mortality, pneumonia, and epilepsy, as well as medical expenditure, length of hospital stay, and ICU care. The increased risk of pneumonia in COPD patients might be explained by the lung changes associated with COPD that lower resistance to lung infections [44]. Although we were unable to demonstrate why COPDe increased the risk of epilepsy in stroke patients, associated nocturnal oxygen desaturation is a plausible explanation [45,46].

Wang et al conducted a nested case-control study and reported that inhaled ipratropium bromide was associated with stroke risk in people with COPD [47]. Lin and his colleagues’ retrospective cohort study reported that pharmacotherapy was associated with stroke risk in patients with COPD [48]. Based on the Taiwan’s National Health Insurance Research Database, the purposes of both these two previous studies were to investigate the influence of COPD medications on stroke risk [47,48]. Different from the previous two studies, our present study evaluated the impacts of COPDne and COPDe on the stroke risk and on the stroke outcomes. The inadequate adjustment for potential confounding factors and the immortal time bias were the main study limitations of the previous study [48]. Besides, the case-control study design could not provide causal inferences for the association between inhaled ipratropium bromide and stroke risk [47]. In addition, short-term follow-up period (3 years) may underestimate the stroke incidence in COPD patients [47]. Finally, mental disorder and hyperlipidemia may be potential confounders for the association between COPD and stroke that were not considered into adjustment in their analysis [47,48].

This study has some limitations. First, we used insurance claims data that lacked information on detailed sociodemographics, lifestyle (such as smoking and alcohol drinking), nutritional level, pulmonary function tests, body mass index, and biomedical measures. Second, though the accuracy of major diagnosis codes from the research database in studies based on these has been accepted by peer reviewers for prominent scientific journals worldwide [11–13,47,48], validity of COPD, stroke, and other codes indicating comorbidities and complications might still be a limitation of this study. Third, our study based on the insurance claims of National Health Insurance program that lack of the information of clinical risk scores for COPD and stroke, such as pneumonia severity index, CURB65, BAP65, or National Institutes of Health Stroke Scale. Therefore, we could not evaluate the impact of these risk score on the risk and adverse events for stroke. In addition, physicians followed standard instructions with using spirometry to diagnose and identify COPD patients in the clinical settings in Taiwan. However, the results of spirometry of COPD patients were not available because the limitation of Taiwan’s National Health Insurance Research Database. The reimbursement claims reported from hospitals and clinics were reviewed strictly by the Ministry of Health and Welfare. Any errors and incorrect information in the insurance reimbursement claims were be withdrawn with punishment and fine. We considered that this limitation may not cause fatal bias to the results of this study. Finally, although we used multivariate adjustment to control several confounders, residual confounding is always possible.

## Conclusion

In conclusion, COPD exacerbation is an important independent risk factor of stroke and post-stroke adverse events. History of COPDe may alert clinicians to these anticipated risks and can affect early decisions about surveillance. Further studies are needed to develop specific strategies to decrease stroke risks and post-stroke adverse outcomes for this patient population.

## Abbreviations

CI: confidence interval
COPD: chronic obstructive pulmonary disease
HR: hazard ratio
ICD-9-CM: International Classification of Diseases, Ninth Revision, Clinical Modification
OR: odds ratio
